# Current Trends in Inpatient Care and In-Hospital Mortality of Cholangiocarcinoma in Germany: A Systematic Analysis between 2010 and 2019

**DOI:** 10.3390/cancers14164038

**Published:** 2022-08-21

**Authors:** Christoph Roderburg, Tobias Essing, Linde Kehmann, Sarah Krieg, Simon Labuhn, Jennis Kandler, Tom Luedde, Sven H. Loosen

**Affiliations:** 1 Department of Gastroenterology, Hepatology and Infectious Diseases, University Hospital Düsseldorf, Medical Faculty of Heinrich Heine University Düsseldorf, 40225 Düsseldorf, Germany; 2 Paracelsus Medical University, Klinikum Nürnberg, 90419 Nürnberg, Germany

**Keywords:** CCA, BTC, biliary tract cancer, chemotherapy, ERCP, epidemiology

## Abstract

**Simple Summary:**

Cholangiocarcinoma (CCA) is a comparatively rare malignant liver disease with an increasing incidence and a high mortality worldwide. Systematic data on epidemiological trends, treatment strategies, and in-hospital mortality of CCA in Germany are missing. The present analysis provides a systematic overview on hospitalized CCA patients in Germany and identifies relevant clinical and epidemiological risk factors associated with an increased in-hospital mortality. These data could help to further improve framework conditions for the management of CCA patients in the future.

**Abstract:**

**Background:** Cholangiocarcinoma (CCA) is a rare malignant disease of the biliary tract with an increasing incidence and a high mortality worldwide. Systematic data on epidemiological trends, treatment strategies, and in-hospital mortality of CCA in Germany are largely missing. However, the evaluation and careful interpretation of these data could help to further improve the treatment strategies and outcome of CCA patients in the future. **Methods:** Standardized hospital discharge data from the German Federal Statistical Office were used to evaluate epidemiological and clinical trends as well as the in-hospital mortality of CCA in Germany between 2010 and 2019. **Results:** A total of 154,515 hospitalized CCA cases were included into the analyses. The number of cases significantly increased over time (*p* < 0.001), with intrahepatic CCA (62.5%) being the most prevalent tumor localization. Overall, in-hospital mortality was 11.4% and remained unchanged over time. In-hospital mortality was significantly associated with patients’ age and tumor localization. The presence of clinical complications such as (sub)acute liver failure, acute respiratory distress syndrome (ARDS), or acute renal failure significantly increased in-hospital mortality up to 77.6%. In-hospital mortality was significantly lower among patients treated at high annual case volume centers. Finally, treatment strategies for CCA significantly changed over time and showed decisive differences with respect to the hospitals’ annual case volume. **Conclusions:** Our data provide a systematic overview on hospitalized CCA patients in Germany. We identified relevant clinical and epidemiological risk factors associated with an increased in-hospital mortality that could help to further improve framework conditions for the management of CCA patients in the future.

## 1. Introduction

Cholangiocarcinoma (CCA) represent a heterogenous group of tumors that can arise anywhere in the biliary tract [1,2]. Based on the anatomic origin, CCA are classified as intrahepatic (iCCA) and extrahepatic (eCCA), with extrahepatic CCAs further subdivided into perihilar CCA (pCCA) and distal CCA (dCCA) [1,2]. In line with their different origin, the subtypes are suggested to have different risk factors, tumor biology, and prognoses [3]. In high income countries, including Germany, CCA represent a comparatively rare disease. Nevertheless, over the last decades, both incidences and mortality rates have continuously risen, which is mainly attributed to the increasing frequency of the iCCAs [1,2]. Due to a silent clinical presentation and its aggressiveness, many patients present in advanced clinical stages, when cure is impossible. Technological advances in all areas of medicine, especially in recent years, have fundamentally changed the way we treat CCA. While extended liver resections have become possible in terms of surgical therapies, novel local ablation techniques such as selective internal radiotherapy (SIRT) have become available in palliative stages, and improved endoscopic procedures have enabled long-term symptom control [1,4,5]. Just recently, combinations of cytotoxic chemotherapy and check-point inhibitors have demonstrated superior tumor control, potentially providing neoadjuvant treatment options enabling long term survival in patients with irresectable disease stages at diagnosis [6].

Although all these advances will significantly improve the clinical management of CCA patients in the future, they also pose new, unprecedented challenges to medical care structures. Further optimization requires a precise understanding of the exact disease epidemiology and, in particular, the knowledge of in which structures and which patients are treated with which outcome. However, such data are hardly available for Germany. In the present study, we used standardized hospital discharge data provided by the Federal Statistical Office of Germany to systematically evaluate recent clinical developments and in-hospital mortality of CCA as well as its influencing factors in Germany between 2010 and 2019. Most importantly, we identified relevant clinical and epidemiological risk factors associated with an increased in-hospital mortality that could help to further the management of CCA patients in future.

## 2. Materials and Methods

### 2.1. Study Design

The present study represents a retrospective analysis of epidemiological trends as well as in-hospital mortality of CCA in Germany. Analyses are based on standardized hospital discharge data provided by the Federal Statistical Office of Germany (Wiesbaden, Germany) from 2010 to 2019. A contract for remote data analysis was signed between the Federal Statistical Office and the University Hospital Duesseldorf in 2020. Due to complete anonymization of patient information, no additional ethics approval was necessary.

### 2.2. Patient Eligibility Criteria and Variables

Identification of the CCA study population was performed via the main diagnosis of the respective hospital stay using the ICD-10 codes C221 (iCCA), C240 (eCCA), C248 (CCA overlapping sites of biliary tract), and C249 (CCA unspecified). Patients with organ complications were identified by the following secondary diagnosis: hepatic encephalopathy (K727), spontaneous bacterial peritonitis (K6500), acute respiratory distress syndrome (J80), sepsis (A02; A20; A26; A32; A40; A41; A42; B37), acute renal failure (N17), acute liver failure (K72.0), liver abscess (K75.0), pneumonia (J13-J18), pulmonary embolism (I260; I269), and ileus (K56). The specific treatment approaches for each patient were identified using the following OPS codes: surgery (5502, 5515), liver transplantation (LT, 55040), chemotherapy (CTX, 8-542, 8-543), transarterial chemoembolization (TACE, 8836ka + 883b10; 8836ka + 883b12; 8836ka + 883b1; 88369a + 883b2x), selective internal radiation therapy (SIRT, 8530a5, 8-530a6, 8-530a8), hyperthermic intraperitoneal chemotherapy (HIPEC, 85460), radiation (8522), photodynamic therapy (PDT, 551341), and radiofrequency ablation (RFA, 551342). The OPS codes 5513f (non-self-expanding stent insertion), 5513h (non-self-expanding stent exchange), 5513m (self-expanding uncovered stent), and 5513n (self-expanding covered stent) were used for complementary analysis of ERC-based treatment options with stents. Additionally, the following clinical and demographical variables were evaluated: sex, age, and federal state of treatment. In-hospital mortality was defined as the proportion of patients whose status of discharged was “death”. With regard to the annual CCA case volume of a respective hospital, we subdivided all treatment centers. We used quartiles of annual case volumes as the basis for this subdivision (low case volume (LCV) hospitals: 1–16 cases/year, medium-low case volume (MLCV) hospitals: 17–35 cases/year, medium-high case volume (MHCV) hospitals: 36–75 cases/year, and high case volume (HCV) hospitals: >76 cases/year). See Table 1 and Table S1 for detailed information.

### 2.3. Statistical Analysis

Statistical analyses were performed via remote data access at the Federal Statistical Office of Germany (Wiesbaden, Germany) using SPSS (IBM Corporation, Armonk, NY, USA) and Excel. Descriptive analyses were performed using cross-tabulations. Comparisons of binary variables (in-hospital mortality) were compared using Pearson’s chi-square test. Changes of dependent and independent variables over time were analyzed by Pearson’s R and linear regression. All statistical tests were two-sided. A *p*-value of *p* < 0.05 was considered statistically significant.

## 3. Results

### 3.1. The Total Number of Inpatient CCA Cases in Germany Is Increasing between 2010 and 2019

**Figure 1:**
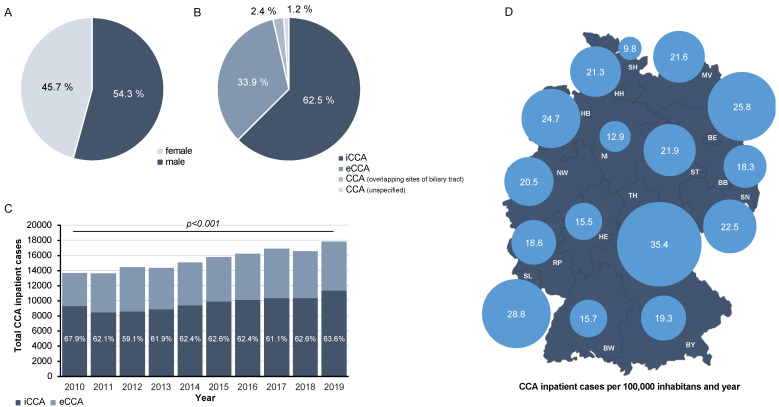
Epidemiological trends of CCA in Germany. (**A**) 54.3% of CCA patients were male. (**B**) iCCA is more common than eCCA. (**C**) There is a significant increase in the overall number of CCA cases between 2010 and 2019. (**D**) The number of inpatient CCA cases per 100,000 inhabitants is highest in Thuringia, Saarland, and Berlin (BB: Brandenburg, BE: Berlin, BW: Baden-Württemberg, BY: Bavaria, HE: Hesse, HB: Bremen, HH: Hamburg, MV: Mecklenburg-Western Pomerania, NI: Lower Saxony, NW: North Rhine-Westphalia, RP: Rhineland-Palatinate, SH: Schleswig-Holstein, SL: Saarland, SN: Saxony, ST: Saxony-Anhalt, TH: Thuringia).

We identified a total of 154,515 individual cases hospitalized for CCA that were included into analyses (Table 1 and Table S1 provide detailed information). Most CCA patients were male (54.3%, Figure 1A). Interestingly, we observed a significant increase in the proportion of male patients over time (2010: 52.1%, 2019: 56.0%, *p* < 0.001, Supplementary Figure S1A). The mean age of the study cohort was 69.6 years (SD: 11.52 years) and significantly increased between 2010 and 2019 (Supplementary Figure S1B). Regarding tumor location, most patients presented with iCCA (62.5%), whereas eCCA (33.9%) was documented less frequently (Figure 1B). While this ratio remained largely consistent over time, we observed a significant increase in the overall number of CCA cases between 2010 (13,680 cases) and 2019 (17,863 cases, Figure 1C). In terms of the geographical region, the number of inpatient CCA cases per 100,000 inhabitants was highest in Thuringia (35.4/100,000 inhabitants and year), Saarland (28.8/100,000 inhabitants and year), and Berlin (25.8/100,000 inhabitants and year, Figure 1D). In contrast, Schleswig-Holstein (9.8/100,000 inhabitants and year) and Hesse (12.9/100,000 inhabitants and year) had significantly lower annual CCA inpatient cases (Figure 1D).

### 3.2. In-Hospital Mortality of CCA in Germany Is Associated with Patients’ Age and the Anatomical Localization of CCA

**Figure 2:**
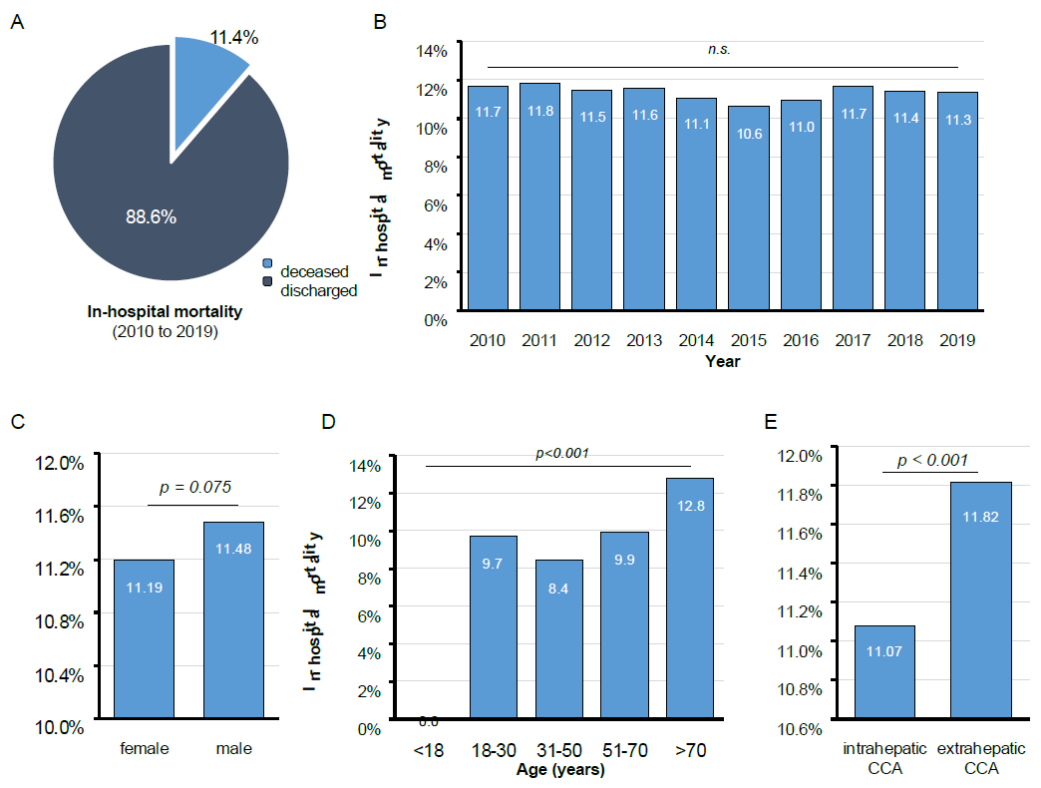
In-hospital mortality of CCA in Germany. (**A**) The overall in-hospital mortality of CCA in Germany between 2010 and 2019 is 11.4%. (**B**) In-hospital mortality does not significantly change during the observation period. (**C**) There is a non-significant trend towards a higher in-hospital mortality among male patients. (**D**) In-hospital mortality significantly correlates with patients’ age and is highest in patients above 70 years. (**E**) In-hospital mortality is significantly higher in patients with eCCA compared to iCCA. n.s.: not significant.

The total in-hospital mortality of CCA in Germany between 2010 and 2019 was 11.4% (Figure 2A). In-hospital mortality did not significantly change during the observation period (Figure 2B). We observed a non-significant trend towards a higher in-hospital mortality among male patients (11.48%) compared to females (11.19%, *p* = 0.075, Figure 2C). In addition, in-hospital mortality significantly correlated with patients’ age and was highest in patients above 70 years (12.8%) and lowest among patients between 31 and 50 years (8.4%, Figure 2D). Interestingly, in-hospital mortality was significantly higher in patients with eCCA (11.82%) compared to iCCA (11.07%, Figure 2E). Of note, in-hospital mortality significantly differed between geographical regions and ranged from 8.9% in Saarland to 13.6% in Lower-Saxony (Supplementary Figure S2A).

### 3.3. Pulmonary, Renal, and Infectious Clinical Complications Increase In-Hospital Mortality of CCA

**Figure 3:**
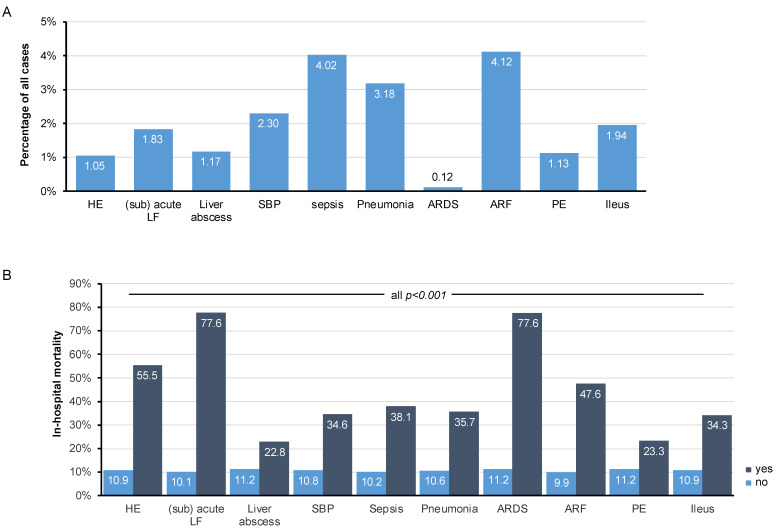
Clinical complications associated with an increased in-hospital mortality of CCA. (**A**) Incidence of common hepatic, infectious, pulmonary, renal, and abdominal complications in CCA patients (HE: hepatic encephalopathy, LF: liver failure, SBP: spontaneous bacterial peritonitis, ARDS: acute respiratory distress syndrome, ARF: acute renal failure, PE: pulmonary embolism). (**B**) All investigated complications lead to a significantly increased in-hospital mortality. In particular, the presence of (sub)acute liver failure or ARDS both dramatically increase in-hospital mortality to over 75%.

Next, we aimed at identifying potential clinical complications that might be associated with an increased in-hospital mortality of CCA. First, we assessed the incidence of common hepatic, infectious, pulmonary, renal, and abdominal complications in CCA patients (Figure 3A). Among these, acute renal failure (ARF, 4.12%) and sepsis (4.02%) were most present, followed by pneumonia (3.18%), spontaneous bacterial peritonitis (2.3%), and ileus (1.94%). Acute respiratory distress syndrome (ARDS) was only observed in a small subset of cases (0.12%). In a second step, we evaluated the impact of each complication on the patients’ in-hospital mortality. Overall, all investigated complications led to a significantly increased in-hospital mortality (all *p* < 0.001, Figure 3B). In particular, the presence of (sub)acute liver failure or ARDS both dramatically increased in-hospital mortality to over 75% compared to only 10.1/11.2% for a patient without liver failure or ARDS, respectively (Figure 3B). In addition, the presence of hepatic encephalopathy (55.5%), ARF (47.6%), and sepsis (38.1%) were key factors associated with an increased in-hospital mortality (Figure 3B).

### 3.4. Chemotherapy Represents the Most Common Treatment Modality for Inpatient CCA Cases in Germany

**Figure 4:**
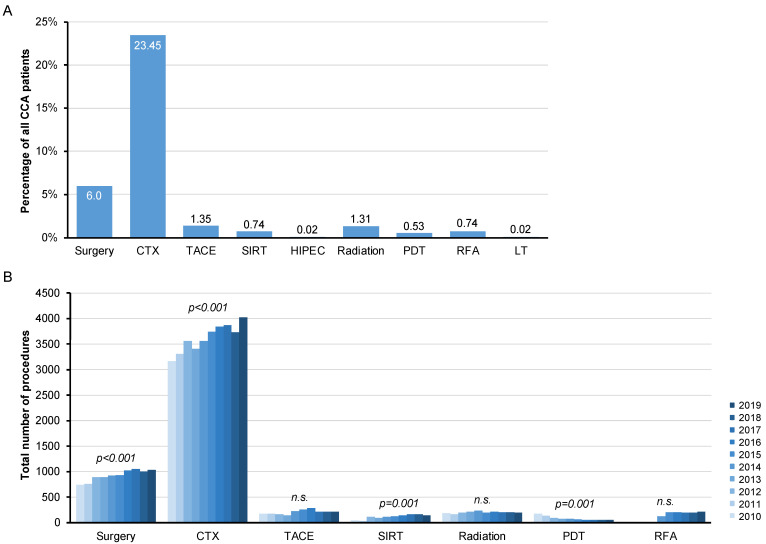
Inpatient treatment paradigms of CCA in Germany. (**A**) Chemotherapy (CTX) is by far the most frequently coded antitumor therapy, followed by surgical resection. Local-ablative therapies such as transarterial chemoembolization (TACE), selective internal radiation therapy (SIRT), or radiation therapy as well as ERC-guided therapeutic approaches including radiofrequency ablation (RFA) and photodynamic therapy (PDT) are less frequently applied. Liver transplantation (LT) or hyperthermic intraperitoneal chemotherapy (HIPEC) are only performed in individual cases. (**B**) Surgical tumor resection and CTX show a significant increase in the number of cases over time. In contrast, the number of local-ablative therapies (TACE, SIRT, RT) remains largely constant over the observation period. n.s.: not significant.

To gain further insights into the treatment landscape of hospitalized CCA patients in Germany, we subsequently analyzed the frequency of antitumoral therapies based on the respective OPS codes that were coded during the hospital stay. At 23.45%, the application of chemotherapy (CTX) was by far the most frequently coded antitumor therapy, followed by surgical resection, which was coded in 6% of all CCA cases (Figure 4A). Local-ablative therapies such as TACE (1.35%), SIRT (0.74%), or radiation therapy (1.31%) were applied less frequently (Figure 4A). Endoscopic retrograde cholangiography (ERC)-guided therapeutic approaches including radiofrequency ablation (RFA, 0.74%) and photodynamic therapy (PDT, 0.53%) were performed in less than one percent of CCA patients (Figure 4A). Further therapies such as liver transplantation (LT, 0.02%) or hyperthermic intraperitoneal chemotherapy (HIPEC, 0.02%) were only used in individual cases (Figure 4A). In a next step, we examined the frequency of application of the individual therapies over the observation period between 2010 and 2019. Interestingly, both surgical tumor resection and CTX showed an increase in the number of cases over time (*p* < 0.001, Figure 4B). In contrast, the number of local-ablative therapies (TACE, SIRT, RT) remained largely constant over the observation period (Figure 4B). With regard to ERC-guided procedures, we observed a significant decrease in the number of PDT cases, while RFA, which was coded for the first time in Germany in 2014, showed an increasing trend (Figure 4B). As eCCA in particular are frequently associated with tumor-induced cholestasis, we finally looked at the number of coded ERC procedures involving stent implantation as a supportive cancer treatment. Overall, 23.7% of patients received stent insertion or exchange during the hospital stay. This number increased significantly between 2010 and 2019 (Supplementary Figure S3A). Looking at the different stent systems available (plastic vs. (un)covered self-expanding mental stent (SEMS)), there was a significant increase in all groups, although it should be noted that the percentage increase in uncovered SEMS, first coded in 2011, was the largest (Supplementary Figure S3B).

### 3.5. In-Hospital Mortality Is Significantly Higher among CCA Patients Treated at Low Annual Case Volume Centres

**Figure 5:**
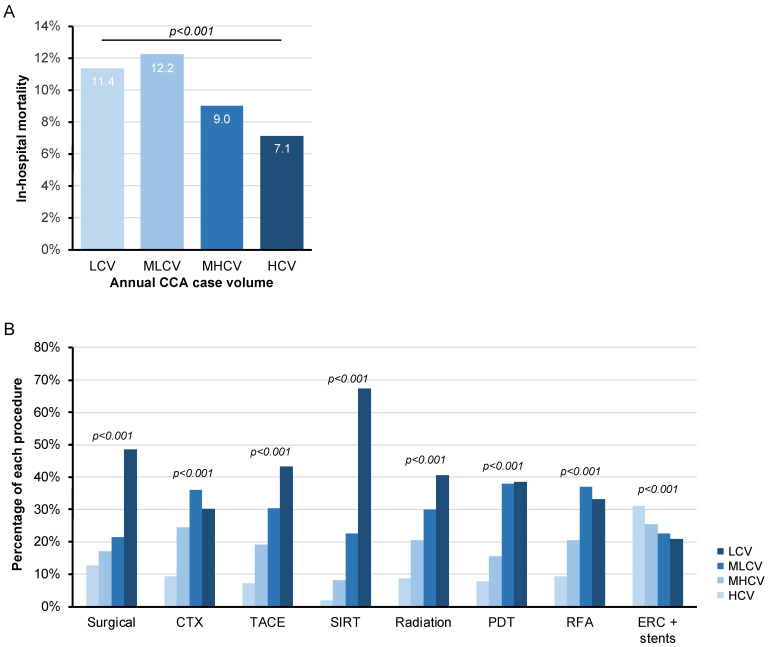
Inpatient care for CCA in terms of the annual case volume. (**A**) In-hospital mortality is significantly lower in MHCV (9.0%) or HCV (7.1%) centers compared to LCV (11.4%) or MLCV (12.2) hospitals. (**B**) The majority of surgical resections (48.5%) or TACE (43.3%), SIRT (67.4%), and radiation therapy (40.6%) procedures are performed at HCV. Most CTXs (36.1%) are administered at MHCV. While most ERC/stent interventions are performed at LVC or MLVC, ERC-guided therapeutic interventions (PDT/RFA) are mainly performed at MHCV (38.0/37.0%) and HCV (38.6/33.2%) centers (LCV: low case volume hospitals: 1–16 CCA cases/year, MLCV: medium-low case volume hospitals: 17–35 cases/year, MHCV: medium-high case volume hospitals: 36–75 cases/year and HCV: high case volume hospitals: >75 cases/year).

We finally analyzed whether the annual CCA case volume had an influence on in-hospital mortality or treatment paradigms of CCA patients. We therefore established four groups of hospitals according to the quartiles of annually treated CCA patients (low case volume (LCV) hospitals: 1–16 CCA cases/year, medium-low case volume (MLCV) hospitals: 17–35 cases/year, medium-high case volume (MHCV) hospitals: 36–75 cases/year and high case volume (HCV) hospitals: >75 cases/year). Interestingly, in-hospital mortality was significantly reduced in MHCV (9.0%) or HCV (7.1%) centers compared to LCV (11.4%) or MLCV (12.2) hospitals (Figure 5A). In addition, we observed significant differences with respect to the various therapeutic interventions between the different caseload categories. As such, the majority of surgical resections (48.5%) or TACE (43.3%), SIRT (67.4%), and radiation therapy (40.6%) procedures were performed at HCV (Figure 5B). In contrast, most CTXs (36.1%) were administered at MHCV. While most ERC/stent interventions were performed at LVC or MLVC, ERC-guided therapeutic interventions (PDT/RFA) were mainly performed at MHCV (38.0/37.0%) and HCV (38.6/33.2%) centers, respectively (Figure 5B).

## 4. Discussion

To our knowledge, our study is the first comprehensive analysis evaluating the presentation, clinical management, and outcome of patients with CCA in Germany. By analyzing a total of 154,515 hospitalized CCA cases, we show that the total number of CCA hospital cases significantly increased between 2010 and 2019. In contrast, in-hospital mortality rates remained constant over the same period of time. In-hospital mortality was significantly associated to the tumor localization and the presence of clinical complications including ileus, COPD, and sepsis. In-hospital mortality was significantly lower among patients treated at high annual case volume centers, which is in line to our finding that chosen treatment strategies showed decisive differences with respect to the hospitals’ annual case volume, highlighting the role of the case volume in guiding patients’ clinical management.

CCA is a highly lethal, epithelial cell malignancy that represents the second most common primary malignancy of the liver [1]. Our finding on increasing CCA-related hospitalization rates during the last decade is consistent with recent reports on globally increasing CCA incidences [2]. In our study, iCCA accounted for the majority of CCA and the increase in CCA hospitalization affected the intrahepatic and extrahepatic forms equally, which is in contrast to previous data [2]. However, when comparing different datasets on CCA epidemiology, it is important to note that the interpretation iCCA/eCCA ratios is hampered by the fact that historical versions of the International Classification of Diseases (ICD) did not include a separate code for pCCA, and previous versions of ICD-Oncology (ICD-O) cross-referenced pCCA to iCCA [7,8,9]. Thus, the lack of a specific code for pCCA may have led to systematic errors, particularly with the miscoding of pCCA as iCCA [8] that may explain the differences between our and previous studies. Just recently, the amended versions of the ICD and ICD-O (ICD-11 and ICD-O-4, respectively) were released, which for the first time feature separate codes to record iCCA, pCCA, and dCCA [10], and will likely lead to more homogeneous and precise data.

Interestingly, we observed regional differences in CCA hospitalization rates between the different German federal states, with higher rates found in the southern/eastern federal states compared to the other states. This finding goes along with previous data describing a tremendous regional heterogeneity in CCA incidences. While differences in CCA rates might be explained by a different distribution of risk factors such as hepatitis virus infection or infections with specific trematodes on a global level, the regional differences in Germany are not so easy to explain. It should be noted that in our analysis we did not record incidences or prevalence per se, but hospital cases. In this respect, it cannot be excluded that the observed regional differences in CCA hospital cases might only reflect differences in local health care structures. On the other hand, differences in lifestyle factors such as an increased consumption of alcohol might be a potential underlying factor, which has been described for the eastern parts of Germany in a different context [11].

In recent years, the therapeutic landscape of CCA has changed considerably. Extended surgical procedures allow tumor resections even in advanced tumors and numerous new local ablative treatment procedures can be offered to CCA patients non amendable to surgery. In our study, however, systematic chemotherapy is by far the most common treatment modality with a continuous and stepwise increase in the last years. This increase is most probably due to the introduction of new indications for systemic treatment in the context of CCA such as adjuvant treatment after complete tumor resection [12] and new second line treatment options [13,14] as well as novel molecular guided treatments [15]. A very similar trend was observed for surgical resection as well as for SIRT, while, interestingly, the number of photodynamic therapies significantly decreased from 2010 to 2019. With respect to the rise of different new treatment modalities, it is important to note that, at least in our analysis, no effect on in-hospital mortality was found. We further analysed an association between the annual CCA case volume and the numbers of specific treatments. Here, our analysis revealed that certain treatment modalities such as SIRT or extended surgery are significantly overrepresented in high volume centres. Interestingly, while these invasive antitumor measures were almost restricted to larger centres, our data revealed a different picture for systemic therapy and endoscopic treatments such as ERC plus stenting, which were performed in nearly identical proportions in small and large hospitals.

Taken together, our study is the first to provide overall CCA-related in-hospital mortality rates in Germany. We found that 11.4% of all hospital cases ended with the patients´ death. The mortality rate remained almost constant between 2010 and 2019. It is important to note that in our analysis, only death during the individual patients´ hospital stay was recorded, which means that no conclusions can be drawn about the mortality or lethality of CCC in Germany. This is important when comparing our results with other analysis on CCA mortality in Germany, Europe, or worldwide [16,17]. These previous analyses showed global increases in mortality from the ICC and declines from ECC for both sexes (ref). Moreover, substantial variations were shown, with the highest rates in East Asia and northern and central Europe [16]. Specifically, in Germany the ICC showed a strong increase in age-standardized mortality rates in the period from 1990 to 2010 (a period not covered by our analyses). The plateau reached hereafter is in line with our data [16]. Our analyses further revealed that in-hospital mortality significantly increased with the patients´ age and that the age group of patients above 70 years showed a significantly increased hospital mortality of 12.8%. A higher prevalence of comorbidities and clinical complications in older patients represents a possible explanation for this observation. We further identified the underlying anatomic tumor localization as a factor associated with hospital mortality, since patients with eCCA demonstrated a by far higher in-hospital mortality than patients with iCCA, most probably reflecting the different clinical presentation of iCCA and eCCA, with eCCA being associated with a more complicative clinical course in many patients. Consistent with previous published data [18], we observed a significantly increased in-hospital mortality at centres with a low annual CCA case volume (see Figure 5). Although this result should be viewed with caution and does not allow generalized conclusions to be drawn, this analysis provides evidence that care in highly specialized centres that offer all therapeutic treatment options could have a favourable effect on the course of the disease in patients with CCA. Even if we cannot attribute the differences in mortality rates between high- and low-volume centres to individual therapeutic modalities, it seems likely that, particularly in the field of surgery, the respective expertise is associated with the patients´ outcome. In Germany, 19,000 patients undergo liver resection each year, 2500 of which are major (30 per million population per year) [19]. A recent analysis revealed an overall in-hospital mortality rate of 5.8 per cent and 10.4 per cent for major resections, which was unchanged in a 6 year period of time [19]. Similar to our data, in this analysis, a significantly lower mortality rate was observed in high-volume centres (45–71 major resections per year) and very high-volume centres (72–171 per year) compared with very low-volume hospitals [19]. Whether such differences between high and low volume centres might lead to regional differences in CCA-related in-hospital mortality cannot be answered from our analysis.

Our study is subject to important limitations. Most importantly, no information on coding quality is available and the database is not subject to systematic quality control between individual hospitals. In particular, difficult codes such as iCCA or eCCA may be recorded differently, which may limit the usability for specific epidemiological questions. CCA patients undergoing surgical tumor resections were identified by the OPS codes 5502 (anatomical (typical) liver resection) and 5515 (operations on gallbladder and bile ducts: excision and resection of diseased tissue in bile ducts) only, which may result in some cases (e.g., patients with distal CCA undergoing pancreatic head resection) not being correctly attributed to the surgical subgroup. In addition, our retrospective database features information on the in-hospital mortality of patients with CCA, but do not allow performing longitudinal analysis on individual patients, which is due to the fact that only anonymized data are provided by the Federal Statistical Office (DESTATIS; Wiesbaden, Germany). Moreover, no detailed information of the specific patients´ history or underlying cause or circumstances of death are available, considerably hampering a detailed analysis on the specific cause of death in the individual patient. Accordingly, to illuminate these factors in more detail, additional prospective randomized controlled trials are needed. These future studies should also include multivariate analyses to further dissect the influence of individual factors on in-hospital mortality.

## 5. Conclusions

Together, our data provide a systematic overview on hospitalized CCA patients in Germany. We identified relevant clinical and epidemiological risk factors associated with an increased in-hospital mortality that could help to further improve framework conditions for the management of CCA patients in the future.

## Figures and Tables

**Table 1:** Characteristics of study population.

	Study Population
Total number of CCA cases	154,515
In-hospital death (total)	17,540
In-hospital mortality rate (%)	11.35
Sex (total, (%))	
male	83,841 (54.26)
female	70,674 (45.74)
Age (Mean and SD)	69.64 (11.15)
Age group (total, (%))	
0–17 Years	21 (0.01)
18–30 years	288 (0.19)
31–50 years	8680 (5.62)
51–70 years	64,469 (41.72)
>70 years	81,057 (52.46)
Federal state (total, (%))	
Baden-Württemberg	16,923 (10.95)
Bavaria	24,584 (15.91)
Berlin	8947 (5.79)
Brandenburg	4521 (2.93)
Bremen	1645 (1.06)
Hamburg	3763 (2.44)
Hesse	9199 (5.95)
Lower Saxony	10,152 (6.57)
Mecklenburg-Western Pomerania	3478 (2.25)
North Rhine-Westphalia	36,384 (23.55)
Rhineland-Palatinate	7501 (4.85)
Saarland	2864 (1.85)
Saxony	9169 (5.93)
Saxony-Anhalt	4929 (3.19)
Schleswig-Holstein	2777 (1.80)
Thuringia	7679 (4.97)
CCA localization (total, (%))	
iCCA	96,631 (62.38)
eCCA	52,348 (33.88)
CCA (overlapping sites of biliary tract)	3699 (2.39)
CCA (unspecified)	1837 (1.19)
Organ Complication (total, (%))	
ARDS	183 (0.12)
ARF	6120 (3.96)
HE	1622 (1.05)
Ileus	2947 (1.91)
Liver abscess	1790 (1.16)
Liver failure	2827 (1.83)
PE	1722 (1.11)
Pneumonia	4760 (3.08)
SBP	3556 (2.30)
Sepsis	6215 (4.02)
Treatment (total, (%))	
CTX	36,233 (23.45)
HIPEC	25 (0.02)
LT	34 (0.02)
PDT	826 (0.53)
RFA	1152 (0.75)
Radiation	2024 (1.31)
Surgery	9266 (6.00)
SIRT	1149 (0.74)
TACE	2087 (1.35)
Annual CCA case volume groups (total, (%))	
LCV (1–16 cases/year)	40,590 (26.27)
MLCV (17–35 cases/year)	37,119 (24.02)
MHCV (36–75 cases/year)	38,547 (24.95)
HCV (>75 cases/year)	38,259 (24.76)

## Data Availability

The underlying data are available at the Federal Statistical Office of Germany.

## Supplementary Materials

The following supporting information can be downloaded at: https://www.mdpi.com/article/10.3390/cancers14164038/s1, Supplementary Figure S1: (A) The percentage of male CCA patients significantly increases over time. (B) The mean CCA patients’ age significantly increases over time. (C) There is a significant difference regarding the distribution of iCCA and eCCA between the different German federal states (BB: Brandenburg, BE: Berlin, BW: Baden-Württemberg, BY: Bavaria, HE: Hesse, HB: Bremen, HH: Hamburg, MV: Mecklenburg-Western Pomerania, NI: Lower Saxony, NW: North Rhine-Westphalia, RP: Rhineland-Palatinate, SH: Schleswig-Holstein, SL: Saarland, SN: Saxony, ST: Saxony-Anhalt, TH: Thuringia). Supplementary Figure S2: (A) In-hospital mortality significantly differs between geographical regions and ranges from 8.9% in Saarland to 13.6% in Lower-Saxony (BB: Brandenburg, BE: Berlin, BW: Baden-Württemberg, BY: Bavaria, HE: Hesse, HB: Bremen, HH: Hamburg, MV: Mecklenburg-Western Pomerania, NI: Lower Saxony, NW: North Rhine-Westphalia, RP: Rhineland-Palatinate, SH: Schleswig-Holstein, SL: Saarland, SN: Saxony, ST: Saxony-Anhalt, TH: Thuringia). Supplementary Figure S3: (A) The total number of CCA patients receiving stent insertion or exchange during significantly increased between 2010 and 2019. (B) There is a significant increase in all groups of stent types, although the percentage increase in uncovered SEMS was highest. Supplementary Table S1. Detailed description of study population from 2010 to 2019.
