# Supplementary material for: Current Trends in Inpatient Care and In-Hospital Mortality of Cholangiocarcinoma in Germany: A Systematic Analysis between 2010 and 2019
Source: Cancers (Basel). 2022 Aug 21;14(16):4038. doi: 10.3390/cancers14164038 (PMC9406726; doi:10.3390/cancers14164038)
Supplement: Supplementary file 1 [file cancers-14-04038-s001.zip › cancers-1825733-supplementary.pdf]

Supplementary Figure Legends

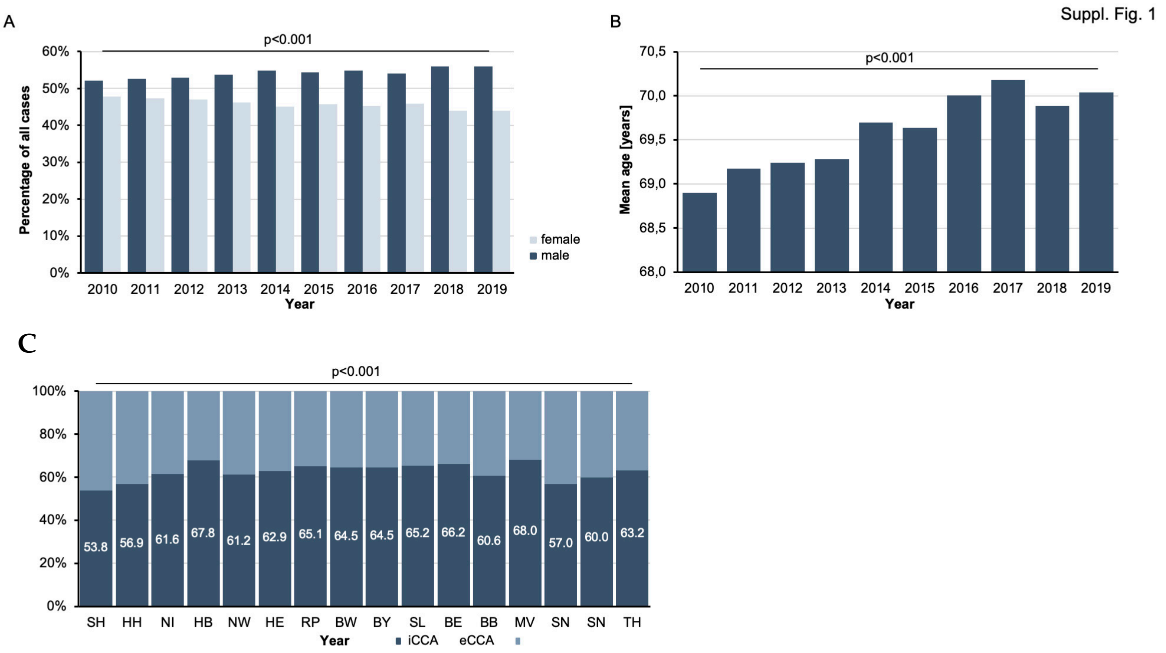

Supplementary Figure S1: (A) The percentage of male CCA patients significantly increases over time. (B) The mean CCA patients' age significantly increases over time. (C) There is a significant difference regarding the distribution of iCCA and eCCA between the different German federal states (BB: Brandenburg, BE: Berlin, BW: Baden-Württemberg, BY: Bavaria, HE: Hesse, HB: Bremen, HH: Hamburg, MV: Mecklenburg-Western Pomerania, NI: Lower Saxony, NW: North Rhine-Westphalia, RP: Rhineland-Palatinate, SH: Schleswig-Holstein, SL: Saarland, SN: Saxony, ST: Saxony-Anhalt, TH: Thuringia).

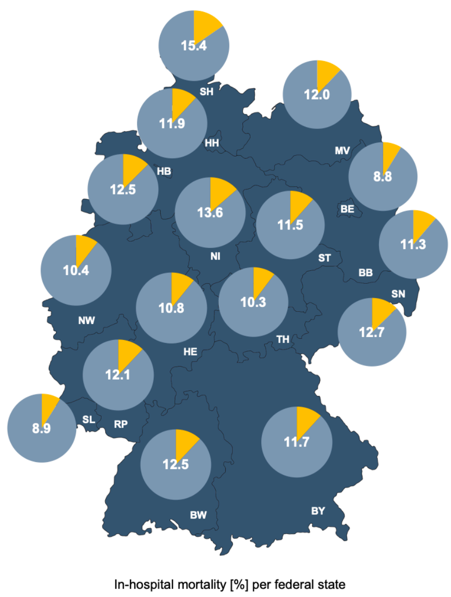

Supplementary Figure S2: (A) In-hospital mortality significantly differs between geographical regions and ranges from 8.9% in Saarland to 13.6% in Lower-Saxony (BB: Brandenburg, BE: Berlin, BW: Baden-Württemberg, BY: Bavaria, HE: Hesse, HB: Bremen, HH: Hamburg, MV: Mecklenburg-

Western Pomerania, NI: Lower Saxony, NW: North Rhine-Westphalia, RP: Rhineland-Palatinate, SH: Schleswig-Holstein, SL: Saarland, SN: Saxony, ST: Saxony-Anhalt, TH: Thuringia).

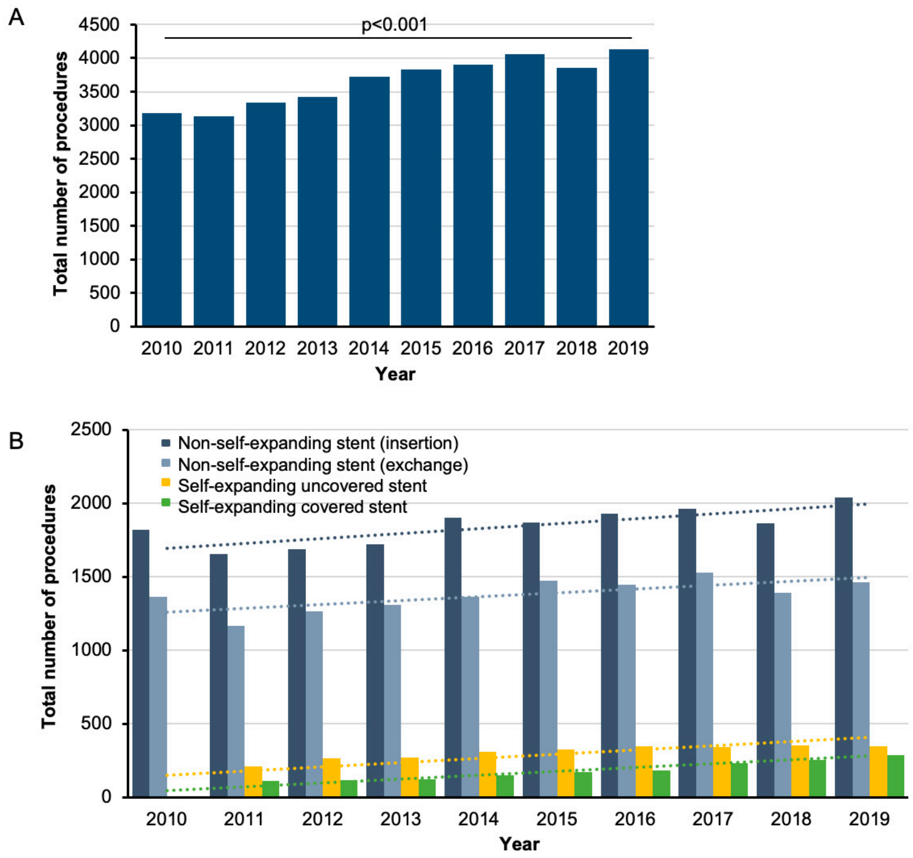

Supplementary Figure S3: (A) The total number of CCA patients receiving stent insertion or exchange during significantly increased between 2010 and 2019. (B) There is a significant increase in all groups of stent types, although the percentage increase in uncovered SEMS was highest.

Supplementary Table S1. Detailed description of study population from 2010 to 2019.

	2010	2011	2012	2013	2014	2015	2016	2017	2018	2019
**Total number of CCA cases**	13,680	13,611	14,453	14,331	15,069	15,802	16,245	16,909	16,552	17,863
In-hospital death [total]	1,598	1,608	1,660	1,657	1,666	1,681	1,779	1,971	1,894	2,026
In-hospital mortality rate [%]	11.68	11.81	11.49	11.56	11.06	10.64	10.95	11.67	11.44	11.34
**Sex** [total]										
Male	7,136	7,164	7,654	7,703	8,279	8,583	8,903	91,35	9,276	10,008
Female	6,544	6,447	6,799	6,628	6,790	7,219	7,342	7,774	7,276	7,855
Age [years, mean]	68.90	69.17	69.24	69.28	69.69	69.64	70.00	70.18	69.89	70.04
**CCA Location** [total]										
Intrahepatic	9,293	8,457	8,550	8,868	9,399	9,886	10,134	10,324	10,364	11,356
Extrahepatic	4,387	5,154	5,903	5,463	5,670	5,916	6,111	6,585	6,188	6,507
**Treatment** [total]										
CTX	3,169	3,311	3,560	3,413	3,563	3,745	3,843	3,873	3,732	4,024
PDT	179	132	91	80	71	61	56	51	50	55
Radiation	188	166	193	215	240	193	215	207	209	198
RFA	0	0	0	0	129	202	205	195	201	220
SIRT	41	33	120	93	120	127	148	164	162	141
Surgery	743	765	894	896	919	934	1,021	1,055	1,000	1,039
TACE	180	178	170	144	222	260	286	216	218	213
Non-self-expanding stent (insertion)	1,817	1,652	1,689	1,718	1,902	1,867	1,931	1,961	1,864	2,041
Non-self-expanding stent (exchange)	1,363	1,166	1,263	1,308	1,364	1,472	1,446	1,529	1,391	1,463
Self-expanding uncovered stent	0	209	264	269	306	323	346	340	353	346
Self-expanding covered stent	0	110	117	122	146	170	179	230	252	287
